# Strategies to Design Technology Promoting Social Participation of People With Dementia and Their Caregivers

**DOI:** 10.1093/geroni/igab046.2252

**Published:** 2021-12-17

**Authors:** Karin Wolf-Ostermann, Jane Flanagan

## Abstract

Community-dwelling people with dementia and their caregivers face increasing challenges to active social participation as the condition progresses. Potential difficulties include disclosing the condition, navigating through available support and sustaining interpersonal relationships. Dementia-friendly support services and interventions targeting caregiving dyads can promote social participation. Interventions serve as a communication channel for the dyads to engage, interact and partake in their community. Technology as a facilitator is gaining momentum; increasing evidence suggests that technological solutions contribute to promoting social health for people with dementia and family caregivers. Patient and public involvement and rigorous evaluations of solutions are needed to ensure successful implementation of dementia-friendly technologies. This symposium, presented as a part of the Marie-Curie Innovative-Training-Network action, H2020-MSCA-ITN, grant agreement number 813196, comprises four pertinent presentations. The first presentation outlines the effectiveness of technological interventions to improve social participation of older adults with and without dementia, and barriers and facilitators these interventions present. The second presentation describes disclosure decisions faced by dyads and Patient and public involvement findings on how an existing empowerment intervention supporting disclosure decision-making can transfer to an online environment. The third presentation reports on findings from a study evaluating a tablet-based activation system designed to engage caregiving dyads in social sessions. The final presentation lifts the focus towards how existing online environments can be adapted through dementia-friendly privacy policy agreements, and thereby support social participation of this user group online. Our discussant, Jane Flanagan, synthesizes the presentations and leads a discussion of future directions for policy and practice.

